# Support during COVID-19 home isolation – clients’ experiences from Finland and Sweden

**DOI:** 10.1186/s12913-026-15231-5

**Published:** 2026-08-05

**Authors:** Camilla Mattjus, Linda Estman, Birgitta Olofsson, Lisbeth Fagerström, Madeleine Blusi

**Affiliations:** 1 Department of Natural and Health Sciences, Åbo Akademi University, Vaasa, Finland; 2 Novia University of Applied Sciences, Vaasa, Finland; 3 Department of Caring and Ethics, University of Stavanger, Stavanger, Norway; 4 Department of Nursing and Department of Diagnostics and Intervention, Orthopedics, Umeå University, Umeå, Sweden; 5 Department of Nursing, Department of Computing Science, Umeå University, Umeå, Sweden

**Keywords:** Home isolation, Support, Patients, COVID-19, Digital support, Digital health, Empowerment

## Abstract

**Background:**

In response to the COVID-19 pandemic, many healthcare clients were required to remain isolated at home after a positive COVID-19 test. Finland and Sweden used different strategies for supporting clients in home isolation.

**Methods:**

This qualitative study explored clients’ experiences of the support provided during home isolation. The participants (*N =* 27) were clients who had been in home isolation during COVID-19 infection in Finland (*n =* 11) and Sweden (*n =* 16). Semi-structured interviews were conducted with participants, and the material was analysed by thematic analysis.

**Results:**

The findings indicate that participants in this study who experienced home isolation described different experiences in Finland and Sweden. In Finland, participants described receiving daily calls from healthcare professionals, whereas in Sweden participants reported being directed to a national web platform where they could access health information or advice if needed. While participants in Finland described extensive contact, they paradoxically experienced more uncertainty. Conversely, participants in Sweden who were directed to a web platform, described navigating their home isolation with greater assurance and empowerment. The findings indicate that it is essential to combine both social and health-related support to effectively endure isolation.

**Conclusion:**

The findings suggest that participants in this study who endured home isolation needed both health-related and social support. Participants appeared to benefit more from a well-established web platform than from daily contacts with healthcare professionals within a new and temporary service structure. Future research should explore the effectiveness of digital support interventions for clients in home isolation during crises, with a focus on client empowerment and satisfaction.

## Introduction

Coronavirus Disease 2019(COVID-19) [1] has impacted people’s lives and health globally. As of August 2023 there had been over 770 million confirmed cases of COVID-19, including over 7 million deaths reported to the World Health Organization (WHO) [2]. For those who had fallen ill or were at risk of falling ill, the situation was frightening. During the pandemic COVID-19 was classified as a dangerous and contagious disease, which greatly concerned those affected. According to the WHO, the most common physical symptoms of COVID-19 are fever, dry cough, and tiredness. Other symptoms include aches and pains, sore throat, diarrhea, headache, loss of smell or taste, skin rashes and/or discoloration of toes or fingers. About 5% of people affected by COVID-19 became critically ill and developed difficulty breathing and/or shortness of breath, chest pain or pressure and/or loss of movement or speech [3]. Persons with underlying medical conditions such as high blood pressure, lung diseases, cardiovascular disease, diabetes or cancer have a higher probability to get critically ill [4]. However, most people (close to 80%) recover from COVID-19 without requiring any hospital stay [5].

The COVID-19 pandemic raised demands for social isolation to prevent dissemination of the virus, which created challenges for those who were isolated. Social isolation can have negative consequences for health, as it is a risk factor for depression, cognitive and physical impairment, violence in close relationships, premature death, heart attack and other outcomes [6, 7]. It can also cause feelings of being meaningless and unneeded and may have negative effects on security and quality of life. Further, loneliness is associated with several physical and mental outcomes, such as elevated systolic pressure and increased risk of heart diseases, and loneliness itself is a risk factor for increased all-cause mortality [8]. People with COVID-19 or their family members/caregivers in isolation should receive support and counseling for symptoms to prevent the negative consequences of COVID-19 [9]. Consequently, they need information regarding what isolation really entails, what directives apply (including self-care advice for those infected), which services are available (e.g. support staff) and how infection control management works (e.g. around protective equipment and aseptic precautions). A need also exists for efficient channels for communication and follow-up of clients’ health conditions.

## Background

Research is difficult to find regarding peoples’ experiences of being in home isolation after testing positive for COVID-19 or suffering from another illness. Earlier studies have shown that participants who lived through a pandemic adapted to the situation and cared for themselves [10]. Moreover, the importance of proper isolation and protective measures for vulnerable people has been emphasized, underscoring the need for studies focused on specific consequences and needs of people that are at greater risk, which ensures their inclusion in health recommendations and considerations of policy makers [11]. Furthermore, it has also been highlighted that isolation significantly distresses mental health [12, 13].

The pandemic brought new needs for healthcare providers, both organisations and healthcare professionals as well as patients. Social and health care during pandemics is governed in Finland by Public Health Act 66/1972 [14] and Communicable Diseases Act 1227/2016 [15], and in Sweden by Health Care Act 2017/30 [16] and Communicable Diseases Act 2004/168 [17]. These laws aim to prevent communicable diseases and their spread as well as their negative consequences on people and society. The laws are applied when the control of infectious diseases is arranged and implemented as well as during planning, control, follow-up, and monitoring of the work. According to these laws, social and health care services are obliged to follow up on and support those who have fallen ill with COVID-19 and to support people in isolation.

### Strategies in Finland and Sweden

Finland and Sweden have comparable publicly funded healthcare systems; however, differences exist in the organisation of primary healthcare and in how responsibility for follow-up of clients is structured. During the study period, Swedish healthcare was organised at the regional level, while Finnish primary healthcare was municipally organised through health centres offering a broad range of services. These organisational differences influenced how support for clients in home isolation was planned and delivered. The process of COVID-19 isolation in Finland started when clients had been exposed to COVID-19 or began to show symptoms, whereupon they received information on the meaning of quarantine and were tested. While waiting for test results, healthcare professionals called those in quarantine at the beginning, during and at the end of quarantine. If the COVID-19 test result came back positive, the quarantine turned into isolation. While the clients were in isolation, the healthcare professionals maintained close contact with them and notified them when they could end the home isolation. If the test results came back negative, the persons could leave quarantine if no symptoms were present.

The process in Sweden started when clients had been exposed to COVID-19 or began experiencing symptoms. They were tested, and if the test turned out positive, clients were placed in quarantine and received a call with information after 1–2 days; they also received written information on recommended measures and the duration of quarantine. Clients added extra days to quarantine on their own initiative and ended quarantine when they had completed the recommended number of days. Notably, it was initially possible to access the national healthcare advisory service, *1177 Vårdguiden*, a Swedish service offering medical advice via both its web platform and telephone service, for guidance on healthcare issues [18] particularly if clients had questions, needed advice, or experienced a deterioration in their well-being.

The two countries’ public health measures may be distinguished by their terminologies and approaches. The biggest differences observed between Finland and Sweden is the amount of contact with clients in home isolation. This occurred daily in Finland, whereas in Sweden it was typically at the beginning of the isolation, but clients also had access to a well-established web platform for further advice. Furthermore, the use of terminology reflected each country’s approach to disease control. In Finland, quarantine could be mandated for those exposed or awaiting test results, while confirmed cases were subject to legally enforced isolation. In Sweden, isolation was used more as a recommendation, with voluntary compliance, and quarantine was rarely applied as a formal measure. However, the implementation of the infection control follow-up posed challenges in terms of the resource capacity of social and healthcare organizations. To prepare for the next pandemic, therefore, it is important to evaluate what worked and what did not work according to the experiences of the clients who experienced the pandemic just passed.

This explorative study at hand is a part of the project PANDA - Innovations for Pandemia Isolation [19], which aimed to identify and explore whether and how the municipalities and regions responsible for infection control follow-up and support for persons in home isolation during the COVID-19 pandemic could be made more effective. The focus was on the Bothnia region in Sweden and Finland. Researchers from Åbo Akademi University and Umeå University collaborated in this project with clients, practitioners working with clients as well as healthcare leaders with responsibility for infection protection. The segment of PANDA described in this article uses interview data, and the focal points are clients’ experiences.

The overall aim of this qualitative study is to explore clients’ experiences of the support provided while they underwent home isolation in Finland and Sweden during the pandemic. This article employes the term *client* to refer to individuals who were both in home isolation and had healthcare contact and uses *home isolation* to denote when clients were ordered to stay home and isolated after testing positive for COVID-19. When referring to the research process, these individuals are also described as participants.

### Materials and methods

This qualitative study used data comprising semi-structured interviews with 27 COVID-19 positive clients in Finland (*n =* 11) and Sweden (*n =* 16) regarding their experiences of support received during a COVID-19 home isolation period (2021). The inclusion criteria were having tested positive for COVID-19, having remained in home isolation according to the guidelines of the health authorities in their country of residence and being over 18 years old. The participants were recruited from urban areas, small towns, and rural municipalities in the regions of Ostrobothnia (Finland) and Västerbotten County (Sweden) through flyers posted at healthcare centres, online announcements, and snowball sampling. Participants were recruited across different local contexts within each region; however, detailed information on specific municipalities was not collected in order to ensure confidentiality. Interested individuals then contacted the research group. The participants included both women (*n =* 17) and men (*n =* 10), and the ages of those interviewed ranged between 22 and 93 years of age (see Table 1).

**Table 1:** Demographic data of the participants

Variable	Finland	Sweden	Total (all participants)
N	11	16	**27**
Age			
Mean	50	47	
Range	31–66	22–93	
Sex			
Female	7	10	**17**
Male	4	6	**10**
Educational degree			
Currently studying	1	1	**2**
Primary school	-	2	**2**
Vocational Degree	5	5	**10**
Bachelor’s degree	3	5	**8**
Master’s degree	1	3	**4**
Doctoral degree	1	-	**1**

### Data collection

Semi-structured interviews were conducted via webcamera or by telephone according to participants’ preferences. The interviews were conducted using an interview guide [20], which included questions, such as the following: What kind of support did you receive during your home isolation/the time you were COVID-19 positive? From whom did you receive support? What kind of information did you receive? What other types of support do you wish you had during your time in home isolation? The interviews were conducted by three researchers (CM, PN & HI). The interviews lasted between 10 and 60 min, were audio recorded and thereafter transcribed verbatim into text, resulting in 161 pages of text in total. The interviews were conducted in Swedish, Finnish and English. Information on participants’ health status and living situation during home isolation was not collected.

### Data analysis

**Fig. 1:**
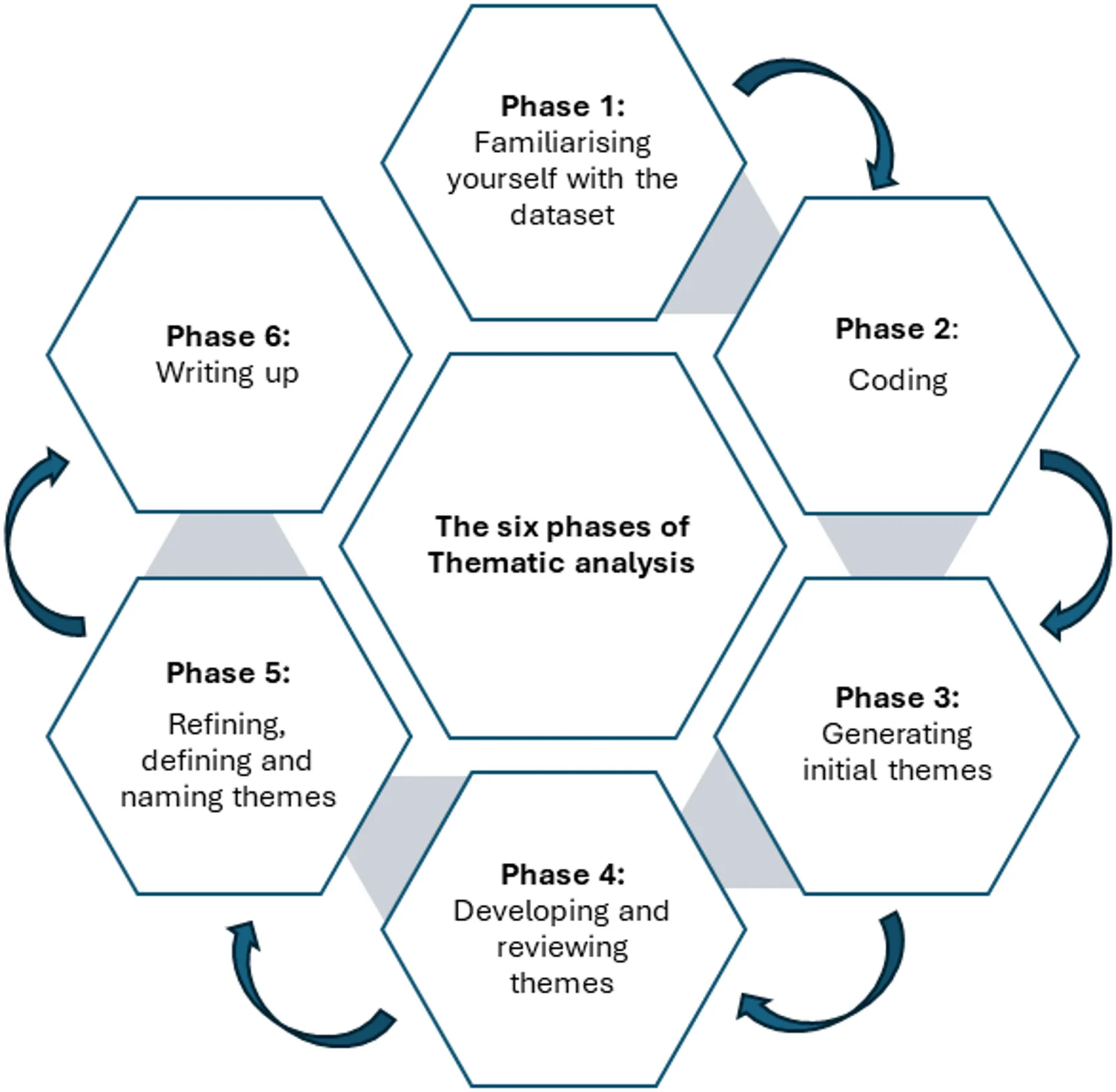
Thematic analysis process based on Braun and Clarke’s six-phase model, illustrated by the authors

The analysis was conducted manually and inductively following Braun and Clarke’s flexible reflexive thematic analysis (TA) framework [21, 22] (see Fig. 1). TA was selected due to its ability to capture both explicit and latent meanings within the data [20]. The transcripts from the interviews underwent thorough reading and re-reading by CM, MB and LE in accordance with phase 1 of the analysis framework [20]. Subsequently, the same three authors collectively analysed the transcribed data, progressing from coding to initial themes and further refinement through phases 2–3 of the analysis scheme. The resulting themes underwent rigorous review and validation through discussions involving the entire research team, corresponding to phases 4–5. Throughout this iterative analysis process, the research team continually revised their analytical steps until they arrived at two comprehensive themes that reflected the data. Concurrently, the writing process (phase 6) proceeded alongside the analysis.

### Ethical considerations

The research strictly followed the guidelines for good scientific practice as outlined by The Finnish National Board on Research Integrity (TENK) [23] and the World Medical Association’s Declaration of Helsinki [24]. Approval for the study was obtained from the Board for Research Ethics at Åbo Akademi University in Finland, as well as from the participating organisations in Finland, and from the Swedish Ethical Review Authority (Dnr 2021 − 01260). The participants were provided with comprehensive verbal and written information regarding the study, ensuring their full understanding. Confidentiality was guaranteed, and the participants were informed of the voluntary nature of their participation, with the option to withdraw at any stage. The participants provided written or verbal informed consent to the interviews.

## Results

The results illustrate the experiences of participants who had been in home isolation because of a positive COVID-19 test. The analysis yielded the two themes, with associated sub-themes (see Table 2), described below: *Trustworthy Information from Predetermined Reliable Sources Gives Confidence and a Sense of Security* and *A Mix of Socially Related and Health-Related Support is Essential for Enduring Isolation*. Quotations are identified using anonymised alphanumeric codes indicating the participant’s country (FI = Finland, S = Sweden) and an individual interview number.

**Table 2:** Overview of themes

THEMES	SUB-THEMES
Trustworthy Information from Predetermined Reliable Sources Gives Confidence and a Sense of Security	Effective Healthcare Communication and Information from Reliable Sources
	Comprehensive Information Builds Confidence and a Sense of Security
	Information About Expanded Self-Care Practices Promote Enhanced Independence
A Mix of Socially Related and Health-Related Support is Essential for Enduring Isolation	Authorities and Social Networks as Vital Pillars of Support
	Balancing Social and Health-related Support for Enduring Home Isolation

### Trustworthy information from predetermined reliable sources gives confidence and a sense of security

This theme, encompassing three sub-themes, offers insights into participants’ experiences regarding the information they received during home isolation, including its sufficiency and their suggestions for additional information to support others in similar situations.

### Effective healthcare communication and information from reliable sources

This sub-theme provides insights into participants’ experiences with the information they received from healthcare while in home isolation, how it was provided, and its content. Participants in both Finland and Sweden received information from healthcare professionals regarding guidelines for COVID-19 and how to act in home isolation, with variations between the two countries in the method of communication. During the isolation period, the Finnish participants were frequently approached for contact and support by healthcare professionals, who telephoned the participant in home isolation daily. Health-related information was given mainly through healthcare calls. The Finnish participants received mostly verbal information from the professional support staff, but some participants mentioned also receiving written health information to some extent.

The nurse called and told me about it and was very caring and everything and then sent me all the information in an email and told me verbally about these isolation regulations. [FI4]

Information was initially given to participants in Sweden via e-mail and during a single telephone call associated with the time of the positive test result. Swedes were advised to use the national health web platform, 1177 Vårdguiden, to seek advice and to contact the healthcare system if they needed further support related to COVID-19. Beyond this initial contact by the professional infection trackers, healthcare professionals in Sweden made no further contacts; instead, Swedes were told to contact healthcare if necessary. Although Swedish participants generally described the web-based service as supportive and sufficient during home isolation, the interviews did not include explicit suggestions for further development of the platform.

They called from infection control and gave information and also sent written information. [S2]

### Comprehensive information builds confidence and a sense of security

This sub-theme sheds light on the participants’ experiences with the information they received, focusing on whether it was sufficient and supportive during home isolation. Participants from both countries were generally satisfied with the information they had received however differences were identified that indicated that experiences to some extent differed between the Swedish and Finnish participants.

Despite frequent contact from healthcare professionals in Finland, there were suggestions for increased frequency of telephone calls and additional support via email. In contrast to the Swedish participants, Finnish participants did not perceive their support as comprehensive, as the participants seemed to lack the former’s confident ability.

But I was really wondering when I really felt bad when should I call somewhere or when should I go to the hospital. It remained unclear to me; of course, it is difficult…but there I would have wished for more support. [FI10]

The Swedish participants felt confident in managing various situations during home isolation thanks to consistent information from multiple sources and the accessibility of 1177 Vårdguiden. They also valued the reliability of information provided through daily press conferences provided by the national public health agency, which aligned with guidance from regional infectious disease controllers. Overall, the well-established digital platform and reliable information sources empowered Swedish participants to make informed decisions and navigate challenges during home isolation with a sense of security.

I feel safe with the information and knew where to turn and what to do if feeling worse…did not need any more support of other things. [S12]

### Information about expanded self-care practices promotes enhanced independence

The last sub-theme addresses suggestions from participants on ways to empower people in home isolation. Receiving trustworthy and useful information in accordance with national guidelines from reliable sources calmed participants in both countries and gave them a sense of security. Even though guidelines differed between Finland and Sweden, participants in both countries trusted their assigned information providers and complied with instructions. Being informed about the guidelines, and obtaining knowledge about COVID-19, isolation regulations and what to do if their health status changed contributed to enduring home isolation. The participants in both countries said that, besides the rich information received, it would have been beneficial to get information about matters that were indirectly related to COVID-19, for example about which services the municipality could have offered to isolated persons besides direct healthcare services. There were also wishes for more contact with other people staying in home isolation and more support in general. The Swedish participants also mentioned that they believed it would have been helpful to receive more information on self-care practices for managing COVID-19 symptoms. They suggested including advice on techniques like positive expiratory pressure breathing exercises, including bottle blowing, and doing toe raises or moving one´s feet to prevent blood clots, especially for individuals who spend much time lying down. Such advice would be good for non-healthcare professionals to receive as well, so that they know what they could do to help themselves.

You could, for example, send direct information by e-mail about what services the municipality can provide in addition to direct care services. So that you can read and think in peace and quiet; do I need any of this? Because all communication except the certificates that we received was verbal. [FI2]

They could have given advice on, well, just blowing in a bottle; maybe if you don’t have compression stockings, you can still do toe raises, not to lie still in bed all day even if you have a fever, but to move around inside. [S10]

### A mix of socially related and health-related support is essential for enduring home isolation

The second theme, consisting of two sub-themes, explores the crucial role of both social and health-related support in helping participants endure isolation.

### Authorities and social networks as vital pillars of support

In this sub-theme, participants share their experiences regarding the importance of support from both authorities and social networks in enduring home isolation. In Finland, the participants felt that they had received social support not only from authorities while in home isolation but also from their own social networks. In addition, other types of support offered by their social networks were mentioned, such as practical support and help with food services. Several Finnish participants had someone who was able to help them out or someone to call through various platforms. A much-appreciated support in both Finland and Sweden was family and friends who stopped by outside the isolated participant’s home to wave and say hello. They followed the recommended physical distancing advice and waved to people outside through the window or from a balcony. Telephone, social media and digital meeting platforms were used to stay in touch with family and friends. Video functions allowing visual contact were considered valuable.

Family came to the door and grandchildren passed by and waved at grandma, and I kept in touch on WhatsApp with my friends. [FI4]

The formal support provided by authorities in Sweden did not include social support; Swedes received social support only through their own social networks. Like the Finnish participants who had help with acquiring food, those in Sweden received assistance with, for example, food deliveries from friends and family who had done their grocery shopping. The Swedes also used home delivery services offered by supermarkets and restaurants.

I received support here at home, both help with food and also someone who checked up on how I was doing at regular intervals now and then. [S8]

### Balancing social and health-related support for enduring home isolation

The Finnish participants expressed that their experience of support from healthcare organisations had, to some extent, been insufficient in terms of organising it. It also seemed that the support given affected the overall experience of whether one felt safe or unsafe. One participant said that one felt significant and very pleased by the support. Several participants also concluded that a social network contributed to a functional support. Some struggled with feelings. Moreover, it was also suggested that perhaps it would be necessary to call some people more frequently to ask about how they are doing, especially for those who are alone.

I probably felt that now that maybe you would need to get support afterwards too, like with long-term symptoms and so on. [FI10]

In Sweden the participants expressed their satisfaction with the organisational support, which was more than enough. The participants felt they had enough knowledge to manage and knew where to seek advice if needed. They also felt the information was mostly clear, but someone expressed that there was not so much information and it was to some extent confusing, and one participant had expressed a feeling of uncertainty, even though satisfied with the support from healthcare. Moreover, some participants expressed concern that people who were alone must have faced difficulties. The Swedish participants highlighted that alternative formats of help would be important for people that are lonely.

But I myself felt a sense of security that I knew where to turn and so on. [S4]

Social support from friends and family was highly valued in Finland. It gave positive energy and contributed to both entertainment and feeling good about knowing one was not forgotten. The social support prevented participants from feeling isolated during home isolation. Family, friends, colleagues, and social media platforms provided social contact. For the Finnish participants, the daily phone calls from healthcare professionals also contributed to a positive social context. Furthermore, the Swedish participants felt that they had received enough support and clear information. They did not wish for any extra support beyond what they had received, and some even pointed out it would have been unnecessary to get more support. All the Swedish participants felt they had knowledge about the rules for home isolation and knew they should turn to the digital platform 1177 Vårdguiden if their health deteriorated or if they had questions.

Participants from both countries experienced that the time spent in home isolation had passed unexpectedly well. Overall, they felt that the support received had been valuable and satisfactory. The importance of having received various forms of support during home isolation was emphasised, and this mix of support had been necessary for them handling it so well. A combination of social support and practical has contributed to successfully enduring the isolation period. Also, the Swedish participants suggested that municipalities could offer additional services for providing food, a suggestion endorsed by the Finnish participants, and in addition to those services providing pharmacological treatment. Moreover, the results reveal that it was a struggle mentally to live with uncertainty knowing that one is COVID-19 positive and not knowing how it would end. A common success factor for enduring and managing in home isolation after tested positive for COVID-19 was the combination of both health-related and socially related support; because health-related support is not enough, social support is also needed to make it through.

## Discussion

This study explored the clients’ experiences of being in home isolation. The findings indicate that the information and support provided in Finland and Sweden differed as did the ways in which information was delivered and support provided to clients. Participants described the support in Finland, which consisted of daily calls, as generally good yet at times insufficient, whereas participants in Sweden described, the web platform 1177 Vårdguiden as sufficient. Normandin et al. found that calls made by volunteers to patients in hospital during the isolation reduced feelings of isolation and provided social interactions to medically isolated individuals [25]. Reflecting on Finland’s strategy, daily calls to clients may have been justified, however, the findings suggest that not only the frequency but also the accessibility of support is important.

Additionally, another study confirmed earlier findings of social challenges, showing that cancer patients, particularly older individuals without children, experienced heightened social difficulties during isolation [26]. This may reinforce the idea that social support plays a crucial role in mitigating the negative effects of isolation. Furthermore, Falvo et al. reveal that lived experiences during isolation demonstrated dissatisfaction and ambivalence at the individual and societal levels due to the epidemic and public health responses, and labelling older adults as a risk group further contributed to societal and self-stigmatisation [27]. In our study, however, older participants did not describe experiences that differed from those of other age groups, but it is important to remember that good intentions to ensure people’s safety can inadvertently lead to stigmatisation and exclusion of certain population groups. Participants in our study generally expressed satisfaction with their experience of being in home isolation, attributing it to the combination of both health-related and social support, which highlights the importance of a holistic approach to support during home isolation that addresses not only health concerns but also individuals’ social and emotional well-being.

These findings may be understood through the theoretical framework of Patient-Centered Care (PCC), which emphasises respecting and responding to individual patient preferences, needs, and values. Effective communication, patient empowerment, and shared decision-making are key elements of PCC, all of which were reflected in the participants’ experiences during home isolation [28]. The findings suggest that health-related support alone may not be sufficient and that social support is also needed to endure home isolation. Additionally, effective healthcare communication has been shown to positively impact patient-centered outcomes among older adults, improving satisfaction, quality of life, and mental health [29]. Although the findings focused on older individuals [29], it may be suggested that effective communication is equally important for younger populations, especially during periods of isolation. Strengthening communication between healthcare professionals and patients, whether through digital tools or direct contact, may therefore be a crucial element of empowering individuals during home isolation.

Prior to analysing the material, it was hypothesised that the clients in Finland might have had a better experience of support due to the almost daily calls to the clients in home isolation, whereas the contact with clients in home isolation was very brief in Sweden. An unexpected finding was that the results contradicted our initial assumption. The Swedish clients believed they had received enough support, as they also knew which place to contact in case something came up, whereas the Finnish clients repeatedly described uncertainty and a desire for more support. It was possible to identify that digital tools were used as means of support. Some clients directly mentioned their use of these tools, whereas others indicated indirectly that a digital tool had been used. A significant difference in digital solutions used was that several clients in Sweden mentioned the platform 1177 Vårdguiden, whereas clients in Finland mentioned phone calls and emails. The findings suggest that the missing link in healthcare support in Finland may be access to a well-established digital platform. Sweden’s well established 1177 Vårdguiden serves as the country’s central hub for healthcare information and services, offering personal e-services and access to health advice via phone [18]. Swedes often turn to this medical service when they have health-related questions. Thanks to its high reliability, it has become a trusted source for medical information and assistance, which fosters safety and a feeling of certainty. By contrast, participants described care in Finland as less immediately accessible, as clients had to wait to get contacted by healthcare, while Swedish clients could handle the matter on their own. Although the findings of this study suggest that access to a well-established digital platform may support clients during home isolation, experiences discussed in this study also indicate that such solutions are not without challenges. Issues related to accessibility, digital literacy, and the need for complementary forms of support should therefore be considered when developing digital support solutions.

When facing challenging situations as people have done in home isolation, it is important to empower clients. Already by 1998 the WHO had made a statement regarding empowerment: ‘Empowerment is a process through which people gain greater control over decisions and actions affecting their health’ [30]. The findings, together with previous research, suggest that digital support could increase accessibility to care [31, 32] and that digital tools offer numerous possibilities for healthcare, including the potential to empower patients. Patient Empowerment has gained significant attention both in practice and theory. Gibson (2001) redefined empowerment as a process of helping individuals assert control over the factors affecting their health. This aligns with the ongoing shift in healthcare where patients are encouraged to take an active role in managing their care. Patient empowerment is a continuous process in which patients collaborate with healthcare systems to become more responsible and involved in their treatment [33]. In the context of this study, the limited availability of digital tools in Finland may have hindered this empowerment process, limiting patients’ ability to engage in self-care practices and gain control over their health during home isolation. However, to genuinely realise the broad potential offered by digital health, strong confidence will be needed in health systems’ ability to protect an individual’s data [34].

A quality improvement project conducted at Singapore General Hospital during the COVID-19 outbreak provides another example of how digital tools can enhance patient empowerment during isolation. Patients were provided with an iPad loaded with the MyCare application, offering curated materials, psychosocial support, and interactive elements. The intervention yielded a positive response from patients, with a high level of satisfaction reported in feedback, and it reduced commonly asked questions, suggesting that the digital platform empowered patients to engage more with their care and feel more connected to their healthcare team. This example reinforces the importance of digital tools in empowering patients, especially during situations of isolation where access to healthcare professionals is limited [35].

The results of this study suggest that a digital solution for support in home isolation can contribute to enabling individuals to gain control, make informed decisions regarding their health and feel empowered. Participants described how Sweden’s digital solution supported them in knowing where to seek advice or assistance when needed, whereas participants in Finland described the absence of such solution as contributing to uncertainty and feelings of powerlessness. These findings suggest that digital tools may empower and enable clients to achieve their health goals [36]. Digital tools and solutions do fall under the umbrella term *digital health* which can be defined as ‘the field of knowledge and practice associated with the development and use of digital technologies to improve health’ [37]. Taken together, these findings suggest that digital tools offer a partial solution regarding how to empower patients. Whilst this is something to consider, disparities can be observed in healthcare in both countries in terms of access to digital tools, which can also explain the health gradient [38].

An important question to consider is whether providing excessive support through daily calls could potentially undermine patient empowerment. Parallels to the concept of helicopter parenting, in which high control leads to low empowerment in children [39], invite further investigation into the impact of overbearing support on patient autonomy, an aspect warranting future research attention. Additionally, this study highlights the significance of examining the role of healthcare infrastructure in shaping patient experiences. Further exploration of this aspect could uncover valuable insights and guide future healthcare practices.

### Method discussion

The study employed TA with an inductive approach to gain a comprehensive understanding of participants’ support experiences during home isolation. This method facilitated a comprehensive exploration of the data, ensuring a refined understanding of clients’ experiences. Following Braun and Clarke’s guidance [22], the themes derived from the analysis were presented in a logical and meaningful order to create a coherent narrative that captured the data’s essence. By adhering to this approach, the study effectively conveyed the complexity of clients’ experiences during home isolation.

To address potential biases in data collection and interpretation, all the researchers participated in the analysis process. The diverse professional backgrounds and experiences of the research team, which include expertise in healthcare practice, enriched the interpretation and minimised the likelihood of bias. The first author, actively working as a nurse, brought direct, practical experience into the analysis, further strengthening the study’s credibility. By engaging multiple researchers with varied experiences, we ensured a comprehensive and balanced approach to data interpretation, fostering a more robust understanding of the findings.

Furthermore, emerging from the methodology employed, the study’s findings include an unexpected result regarding experiences in Sweden compared to Finland. This unexpected outcome emphasises the importance of the methodology used. The employed methodology facilitated exploration of clients’ support experiences, and the absence of a well-established digital health platform in Finland emerged as a significant factor. This unexpected result highlights the need for future research to consider the role of healthcare infrastructure in shaping client experiences, demonstrating the complexities inherent in healthcare research.

### Study strengths and limitations

The study’s strengths include a diverse recruitment strategy, including online fliers, posters in healthcare centres to contact individuals representing the sample. Ethical considerations were addressed through the provision of both verbal and written information to participants. Also, the collaboration between researchers from different universities and participants from different countries enriched the study with diverse perspectives and ensured relevance to real-world healthcare practices.

The study’s limitations may be due to its reliance on participant recall, which could introduce bias. The participants were required to remember past support experiences during home isolation, potentially impacting data reliability. The study’s narrow focus on specific regions and strict inclusion criteria may limit generalisability to other populations or contexts. Recruitment via online fliers and posters in healthcare centers could introduce selection bias, favoring individuals with better access to healthcare.

## Conclusion

In response to the COVID-19 pandemic, many clients have faced isolation at home, revealing varying support experiences between Finland and Sweden. Participants in both countries emphasised the importance of timely information and accessible support. In Finland, support consisted mainly of daily calls, whereas Sweden made the best use of its well-established web platform. However, despite the efforts, some participants in Finland still felt left alone without sufficient digital support. In Finland, a significant gap exists due to the absence of a digital support solution, impacting client satisfaction during home isolation. To address this gap and enhance client empowerment and satisfaction, developing and implementing a tailored digital support solution is imperative. Digital tools have the potential to improve client experiences and overall well-being during isolation by providing information, facilitating communication with healthcare professionals and offering a sense of control. Future research should focus on evaluating the effectiveness of different support interventions, particularly digital solutions, in crisis contexts.

## Data Availability

The datasets generated and/or analysed during the current study, including interview transcripts and related materials, are available from the corresponding author upon reasonable request.

## Abbreviations

COVID-19: Coronavirus Disease 2019
PCC: Patient-centred care
PANDA: Innovations for Pandemia Isolation
TA: Thematic Analysis
TENK: The Finnish National Board on Research Integrity
WHO: World Health Organization
